# PD-1 inhibitors plus nab-paclitaxel-containing chemotherapy for advanced gallbladder cancer in a second-line setting: A retrospective analysis of a case series

**DOI:** 10.3389/fonc.2022.1006075

**Published:** 2022-11-16

**Authors:** Sirui Tan, Jing Yu, Qiyue Huang, Nan Zhou, Hongfeng Gou

**Affiliations:** Department of Abdominal Cancer, West China Medical School, Cancer Center, West China Hospital, Sichuan University, Chengdu, China

**Keywords:** advanced gallbladder cancer, efficacy, PD1, nab-paclitaxel, chemotherapy

## Abstract

**Background:**

Gallbladder cancer (GBC) is a fatal cancer, and the efficacy of the current standard second-line chemotherapy for GBC is limited. Novel therapies need to be explored. This retrospective analysis was aimed to investigate the outcomes of patients treated at West China Hospital with PD-1 inhibitors combined with nab-paclitaxel-based chemotherapy (nab-paclitaxel monotherapy or nab-paclitaxel plus other cytotoxic agents) in a second-line setting.

**Methods:**

Between April 2020 and May 2022, the patients with advanced GBC receiving PD-1 inhibitors combined with nab-paclitaxel-based chemotherapy after resistance to first-line gemcitabine-based chemotherapy at West China Hospital were retrospectively screened.

**Results:**

Eleven patients were included, and all received gemcitabine-based chemotherapy as first-line therapy. Eight patients underwent next-generation sequencing (NGS), and all had microsatellite stability (MSS) and a low tumor mutation burden (TMB). Six patients were negative for PD-L1 expression and one patient was positive for PD-L1. Therapeutically relevant genetic alterations were not found. All patients received PD-1 inhibitors in combination with nab-paclitaxel-based chemotherapy as second-line therapy. Pembrolizumab was administered in 3 patients, and sintilimab was administered in eight patients. One patient had no measurable target lesion. Complete response (CR) was observed in one (10.0%) patient, partial response (PR) in four (40%) patients, and stable disease (SD) in four (40%) patients. The median progression-free survival (PFS) was 7.5 (95% CI: 2.5-12.5) months, and the median overall survival (OS) was 12.7 (95% CI: 5.5-19.9) months. The adverse events (AEs) were manageable.

**Conclusion:**

Our results suggest that PD-1 inhibitors combined with nab-paclitaxel-based chemotherapy as second-line therapy for advanced GBC might be a potential treatment and deserves further evaluation.

## Introduction

Gallbladder cancer (GBC) is a rare disease among all tumors but is considered a common biliary tract carcinoma (BTC) (1). Its incidence ranks sixth among gastrointestinal tract tumors and shows significant geographical differences globally. Compared to that in other countries, the incidence of GBC in China is at an intermediate level, while areas of high prevalence include Chile, Poland, northern India, Japan, and Israel (2). To date, surgery is the only way to cure localized GBC, but most patients are diagnosed at unresectable or metastatic stages because of the insidious onset and asymptomatic characteristics of GBC. Even after radical surgery for GBC, the recurrence rate is high within one year, and the 5-year survival rate of GBC is approximately 5%-15% (3). Presently, chemotherapy is most commonly used for advanced GBC but shows an unsatisfactory prognosis.

The standard first- and second-line chemotherapy regimens for advanced BTC are gemcitabine plus cisplatin and FOFLOX, respectively (4, 5). The median overall survival (OS) was prolonged by only 0.9 months compared to active symptom control in standard second-line FOFLOX chemotherapy, providing very limited benefit (5). Multiple phase II and phase III trials have shown that novel targeted agents for fibroblast growth factor receptor 2 (FGFR2) fusions and isocitrate dehydrogenase-1 (IDH1) mutations as second-line therapy for patients with such abnormalities have obtained good results (6–8). However, patients with FGFR2 fusions and IDH1 mutations were predominantly patients with intrahepatic cholangiocarcinoma, and none of the patients with GBC were included in these studies. To date, specific biomarkers for GBC are poorly defined. HER2/neu amplification is detected in 12–15% of GBC patients (9). Only small patient cohorts have been used to investigate the use of HER2-directed targeted therapies in advanced GBC, providing limited data (10–12). Poor treatment outcomes with standard chemotherapy and a lack of targeted GBC drugs have prompted research into new effective treatment strategies.

The effect of nab-paclitaxel on second-line treatment in patients with advanced GBC has not been studied in large prospective studies. However, nab-paclitaxel has shown promising effects in patients with advanced BTC in early clinical trials. In phase II prospective trials, nab-paclitaxel-based chemotherapy (gemcitabine, cisplatin, and nab-paclitaxel or nab-paclitaxel and gemcitabine) showed favorable outcomes in the first-line treatment for advanced BTC (13, 14). Moreover, in a small series, nab-paclitaxel and capecitabine appeared to have a biological activity by controlling BTC with a disease control rate (DCR) of 81% (9/11), positively affecting survival in second-line treatment (15). A retrospective study suggested the clinical benefit of nab-paclitaxel monotherapy in prolonging OS in advanced GBC patients after failure of the first-line gemcitabine and platinum and the second-line FOLFOX-4 therapy (16). Overall, these data indicate that nab-paclitaxel monotherapy or nab-paclitaxel-based combination chemotherapy can provide a response in advanced GBC patients. Immunotherapy has also been extensively studied in advanced BTC. The TOPAZ-1 phase 3 trial even extended the guidelines and established durvalumab (programmed cell death 1-ligand 1(PD-L1) inhibitor) and gemcitabine–cisplatin as the first-line treatment internationally, on the basis of an improved OS (12.8 months *vs*.11.5 months; HR 0.80, 95% CI 0.66–0.97; P ¼ 0.021) compared with placebo and gemcitabine–cisplatin arm (17). Multiple trials of PD-1 inhibitors plus chemotherapy are ongoing (NCT04003636, NCT03111732, NCT03486678). There are no reports of PD-1 inhibitors combined with nab-paclitaxel-based chemotherapy for the second-line treatment of GBC, athough this combination has been widely used to treat other cancers (18–20). Our institutional experience revealed some patients with unexpected and notable benefit to this combination following progression while on gemcitabine-based chemotherapy. In our report, we collected medical records and retrospectively analyzed the outcomes of gemcitabine-refractory GBC patients treated with PD-1 inhibitors combined with nab-paclitaxel-based chemotherapy as second-line therapy with the aim of providing an exploratory role for future prospective clinical trials.

## Patients and methods

After obtaining approval from the ethics committee on biomedical research, West China Hospital of Sichuan University, from April 2020 to May 2022, patients with pathologically confirmed GBC treated with PD-1 inhibitors plus nab-paclitaxel-based chemotherapy as second-line therapy at West China Hospital were identified. Patients were included if they received first-line gemcitabine-based chemotherapy. Patients were excluded if they received immunotherapy or nab-paclitaxel-based chemotherapy as first-line treatment. Any regimen of nab-paclitaxel-based chemotherapy administered as second-line therapy for advanced GBC was allowed, including single-agent and combination treatments. There were no restrictions on the type of PD-1 inhibitor used. Treatment options were related to the patient’s individual situation. Patients were fully informed of the treatment options available and the advantages and disadvantages of each option before treatment. Patients signed informed consent forms before receiving PD-1 inhibitors in combination with nab-paclitaxel-based chemotherapy. Sex, age, site of metastases, Eastern Cooperative Oncology Group (ECOG) performance status, histology, disease stage, therapeutic approach, clinical efficacy, and adverse effects were reviewed from the medical records. The tumor mutational burden (TMB), microsatellite instability (MSI) status, and driver gene mutations were tested by next-generation sequencing (NGS). Immunohistochemical (IHC) staining was used to determine PD-L1 expression.

The tumor responses were assessed by computed tomography (CT) scans according to the Response Evaluation Criteria in Solid Tumors (RECIST) version 1.1. Tumor response was categorized as complete response (CR), partial response (PR), stable disease (SD), and progressive disease (PD). The objective response rate (ORR) included evaluations of the patients with CR and PR, and the DCR included the evaluations of the patients with CR, PR, and SD. OS was calculated from the administration date of second-line combined therapy to the date of death of the last follow-up. The patients still alive at the last follow-up were censored for OS. Progression-free survival (PFS) was calculated from the date of combined treatment to the date of progression, death, or last follow-up. The patients alive and without progression were censored for PFS. The last follow-up date was 2022-07-22. PFS and OS were estimated using the Kaplan‐Meier method and compared by the log-rank test.

Adverse effects (AEs) and safety assessments were recorded in the electronic medical records. The investigators used the Common Terminology Criteria for Adverse Events (CTCAE) version 5.0 to grade the AEs from 1 to 5.

## Results

### Patient characteristics

The baseline characteristics of all the patients are summarized in **Table 1**. There were 7 females and 4 males, with a median age of 57 (IQR: 53-67) years. Nine patients (81.8%) had an ECOG performance status of 0, and two patients (18.2%) had an ECOG performance status of 1. Before second-line treatment, all patients had TNM stage IV adenocarcinomas, and 2 (18.2%) patients had poorly differentiated GBC. Patients had metastatic cancer in the peritoneum (9/11, 81.8%), lymph nodes (8/11, 72.7%), and liver (7/11, 63.6%). Regarding prior treatments, recurrence occurred in 4 (36.4%) patients after radical surgical resection, and 3 (27.3%) patients had palliative surgery. All patients received first-line therapy with gemcitabine-based chemotherapy. Three patients were treated with the gemcitabine/capecitabine (GX) regimen, and 8 were treated with the gemcitabine/cisplatin (GP) regimen in the first-line setting. Three patients underwent radical surgery and received adjuvant therapy with the GX regimen, but all 3 patients relapsed within 6 months after surgery. The baseline median CA19-9 level was 66 (IQR: 8.5-484.0) U/ml. Eight patients underwent NGS and were microsatellite stable and had a low TMB. The median TMB was 6 Muts/Mb (IQR: 2.3-12.9). None of these patients had therapeutically relevant genetic alterations. Six patients showed negative PD-L1 expression, and one patient was positive for PD-L1. Eight (8/11, 72.7%) patients received PD-1 inhibitors in combination with nab-paclitaxel monotherapy, and three patients (3/11, 27.3%) received PD-1 inhibitors plus nab-paclitaxel-based combination chemotherapy. Of the 11 patients, 3 were treated with pembrolizumab, and 8 were treated with sintilimab. The details about the second-line regimens, NGS results, the expression of PD-L1, and duration of treatment are described in **Table 2**.

**Table 1:** Baseline patient characteristics.

Characteristics	Number (%)
Sex	
Female	7 (63.6)
Male	4 (36.4)
Age, years (median, IQR)	57 (53-67)
ECOG performance status	
0	9 (81.8)
1	2 (18.2)
Tumor stage	
IV	11 (100)
Histology	
Adenocarcinoma	11 (100)
Differentiation	
Moderately poorly	2 (18.2)
Moderately	3 (27.3)
Poorly	2 (18.2)
Unsure	4 (36.4)
Site of metastases	
Lymph node	8 (72.7)
Peritoneal metastasis	9 (81.8)
Liver	7 (63.6)
Ovary	2 (18.2)
Adrenal gland	1 (9.1)
Previous antitumor therapy	
Radical surgery resection	4 (36.4)
Palliative surgery	3 (27.3)
Systemic chemotherapy	11 (100)
CA19-9 (decreased)	7 (63.6)
PD-L1 expression	
Positive	1 (9.1)
Negative	6 (54.5)
NA	4 (36.4)
MSI	
MSS	8 (72.7)
NA	3 (27.3)
TMB, Muts/Mb (median, IQR)	6 (2.3-12.9)
TMB-L	8 (72.7)
NA	3 (27.3)

ECOG, Eastern Cooperative Oncology Group; CA19-9, cancer antigen 19–9; MSI, microsatellite instability; NA, not available; TMB, tumor mutation burden; PD-L1, programmed cell death ligand 1.

**Table 2:** Summary of the therapeutic strategies, NGS results, PD-L1 expression and clinical responses.

Patient	Immunotherapy^a^	Concurrent therapy	PD-L1	TMB (Muts/Mb)	MSI status	PFS (months)	OS (months)	Best response
1	Sintilimab	Nab-Paclitaxel+S-1	–	3.9	MSS	7.5	12.7	PR
2	Sintilimab	Nab-Paclitaxel	–	3.4	MSS	6.8	12.4	PR
3	Pembrolizumab	Nab-Paclitaxel	–	7.3	MSS	11.0	14.8+	SD
4	Pembrolizumab	Nab-Paclitaxel	–	0.0	MSS	18.4+	18.4+	PR
5	Sintilimab	Nab-Paclitaxel	NA	NA	NA	7.7+	7.7+	SD
6	Sintilimab	Nab-Paclitaxel	–	NA	NA	3.5	6.1	non-CR/non-PD^b^
7	Pembrolizumab	Gemcitabine + Cisplatin+Nab-Paclitaxel	NA	NA	NA	3.6	5.2	SD
8	Sintilimab	Nab-Paclitaxel	TPS 5%, CPS 10	1.9	MSS	4.0+	4.0+	CR
9	Sintilimab	Nab-Paclitaxel	NA	8.6	MSS	2.5	2.5+	PD
10	Sintilimab	Nab-Paclitaxel	NA	15.0	MSS	1.7+	1.7+	SD
11	Sintilimab	Cisplatin+Nab-Paclitaxel	–	14.3	MSS	1.5+	1.5+	PR

PR, partial response; SD, stable disease; CR, complete response; PD, progressive disease; MSS, microsatellite stability; NA, not available; TPS, tumor proportion score; CPS, combined proportion score.

a Sintilimab and pembrolizumab are PD-1 inhibitors.

b The patient did not have target lesions therefore she was evaluated as non-CR/non-PD according to RECIST 1.1.

### Efficacy

**Figure 1:**
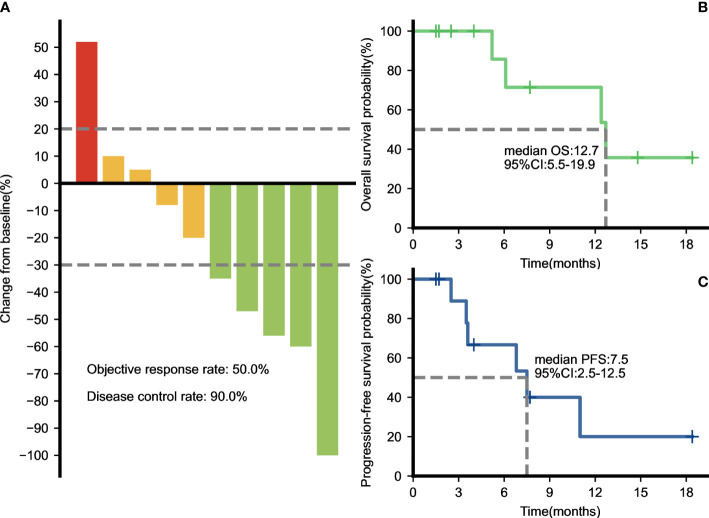
Therapeutic efficacy of PD-1 inhibitors plus nab-paclitaxel-based chemotherapies. **(A)** Maximum percentage change in the sum of the diameters of the target lesions from baseline. The overall survival **(B)** and progression-free survival **(C)** curves of the entire cohort.

**Figure 2:**
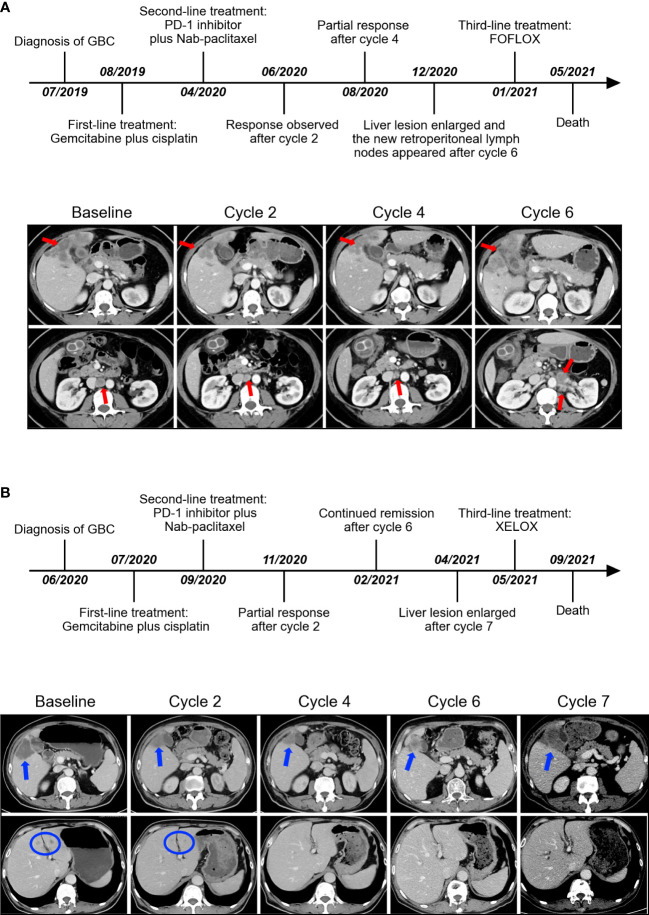
The timeline of the entire clinical course and images presenting the second-line therapeutic responses in representative partial-response patients **(A, B)**. Arrows and circles indicate the position of lesions.

**Figure 3:**
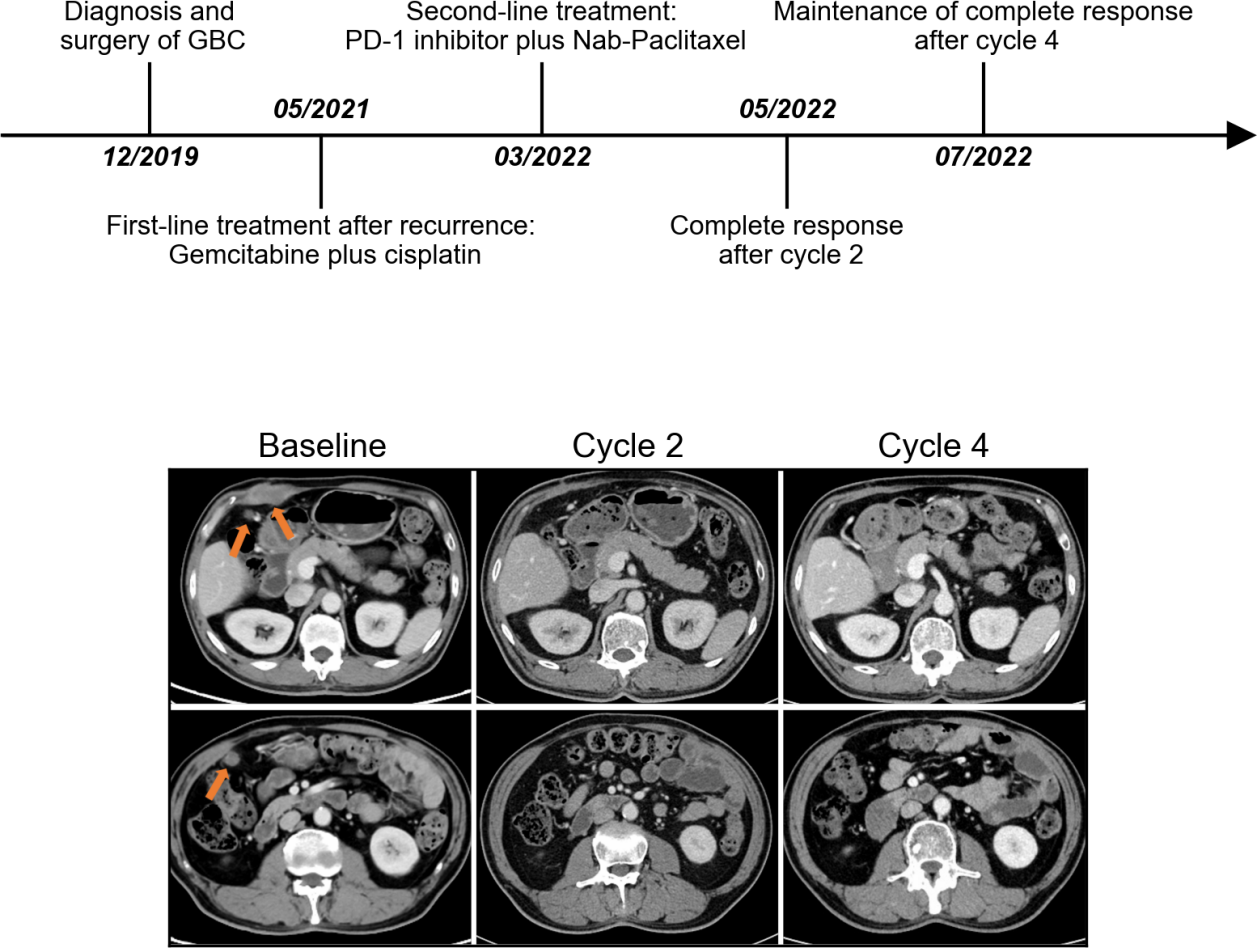
The timeline of the entire clinical course and images presenting the second-line therapeutic responses in a representative complete-response patient. Arrows indicate the position of lesions.

The median duration of treatment with PD-1 inhibitors in combination with nab-paclitaxel-based chemotherapy was 5.3 months. After the initiation of second-line therapy, 7 patients (63.6%) showed decreased CA19-9 levels. All patients underwent radiological evaluations. One patient had no measurable lesions. Among 10 patients evaluable for efficacy according to RECIST criteria, 7 patients (70%) showed a decrease in the tumor size from baseline (**Figure 1A**). PR was observed in four (40.0%) patients (**Figure 2**), and CR was observed in one (10%) (**Figure 3**), which corresponded to an ORR of 50%. The DCR was 90%, including 4 (40%) patients who had SD as the best response (**Table 3**). The median PFS was 7.5 (95% CI: 2.5-12.5) months (**Figure 1B**), and the OS was 12.7 (95% CI: 5.5-19.9) months (**Figure 1C**). Five patients are currently still on therapy, and one patient is receiving backline palliative treatment.

**Table 3:** Clinical antitumor activity.

Therapeutic response assessment	Evaluable patients
Complete response	1 (10.0)
Partial response	4 (40.0)
Stable disease	4 (40.0)
Progressive disease	1 (10.0)
Confirmed objective response rate	5 (50.0%)
Confirmed disease control rate	9 (90.0%)
Median overall survival, months (95% CI)	12.7 (5.5-19.9)
Median progression-free survival, months (95% CI)	7.5 (2.5-12.5)

### Safety

All patients (100%) developed treatment-related AEs. The most frequent adverse events were hair loss (100%), neutropenia (72.7%), and anemia (45.5%). The common grade III/IV AEs included neutropenia and anemia. There were no treatment-related deaths at the time of analysis (**Table 4**). After careful treatment, most AEs were tolerated and well controlled.

**Table 4:** Treatment-related adverse events in 11 patients.

Treatment-related event, n (%)	Any grade	Grade 1–2	Grade 3	Grade 4
Fatigue	3 (27.3)	3 (27.3)	0	0
Vomiting	4 (36.4)	4 (36.4)	0	0
Skin rash	1 (9.1)	1 (9.1)	0	0
Peripheral neurotoxicity	4 (36.4)	4 (36.4)	0	0
Hair loss	11 (100)	11 (100)	0	0
Neutropenia	8 (72.7)	4 (36.4)	2 (18.2)	2 (18.2)
Anemia	5 (45.5)	3 (27.3)	2 (18.2)	0
Thrombocytopenia	3 (27.3)	2 (18.2)	1 (9.1)	0
Fever	2 (18.2)	2 (18.2)	0	0
Bilirubin elevation	2 (18.2)	1 (9.1)	1 (9.1)	0
ALT or AST elevation	4 (36.4)	4 (36.4)	0	0
Decreased weight	2 (18.2)	2 (18.2)	0	0
Hypothyroidism	1 (9.1)	1 (9.1)	0	0

## Discussion

Patients with advanced GBC have limited treatment options and a poor overall prognosis, so new therapeutic approaches are needed. Only patients with GBC were included in our report. To the best of our knowledge, this is the first report to specifically analyze the treatment results of gemcitabine-refractory GBC patients treated with PD-1 inhibitors in combination with nab-paclitaxel-based chemotherapy. We observed an ORR of 50%, a DCR of 90%, an mPFS of 7.5 months, and an mOS of 12.7 months, which were much better than those of the GBC patients treated with a single PD-1 inhibitor or traditional chemotherapy, and the patients treated with this combination had a manageable toxicity profile. The evaluation of GBC patients in our report indicated that a combination strategy of anti-PD-1 with nab-paclitaxel-based chemotherapy as second-line therapy had favorable efficacy.

Second-line chemotherapy or immune checkpoint inhibitor (ICI) monotherapy for advanced BTC has limited benefits. In the ABC-06 study, the FOLFOX treatment group included only 17 patients with GBC, and the subgroup analysis showed that there was no obvious benefit of the FOLFOX regimen for GBC patients (5). Due to the limited efficacy and high incidence of grade 3-4 AEs, standard second-line FOLFOX chemotherapy is not widely accepted in clinical practice. In addition, other potential second-line chemotherapies have similarly limited efficacy in GBC (21). In previous prospective or retrospective studies, the use of ICI monotherapy as a second-line treatment for BTC patients, including GBC, showed a modest efficacy with an ORR of 3%-22%, mPFS of 1.8-3.68 months, and mOS of 4.3.2-14.2 months, which did not demonstrate an outstanding advantage over chemotherapy (5, 22–25). In a single-arm, multicenter phase 2 study of nivolumab, an ORR of 15% (2 of 13) in GBC was observed, which was lower than that of other forms of BTC (26). Based on these limited results, chemotherapy or ICI alone has limitations for the backline treatment of GBC.

Combined regimens of PD-1/PD-L1 inhibitors with other therapies for advanced BTC have been increasingly practiced in clinics (27–30). Some small-size phase I and phase II clinical studies have shown that the efficacy of dual ICIs and ICIs with targeted therapy in biliary tract tumors was also very limited; notably, there were very few patients with GBC in these studies (27, 30). However, ICIs combined with chemotherapy has emerged as a promising strategy for BTC (31). In the first-line phase 3 TOPAZ-1 study, the use of durvalumab plus gemcitabine and cisplatin first demonstrated a statistically significant prolonged OS in BTC patients (17). The subgroup analysis showed that there was no obvious benefit for GBC patients in the TOPAZ-1 study (31). Most studies did not perform an anatomical subgroup analysis due to the small number of patients. The genomic alterations in GBC are very different from those in cholangiocarcinoma (32). The findings derived from clinical trials for BTC cannot be automatically extended to the GBC population. There are very few studies on immunotherapy for gallbladder cancer alone. In a real-world study, 31 GBC patients received ICIs plus lenvatinib in first- or second-line therapy. The ORR was 32.3%, the mPFS was 5.0 months and the mOS was 11.3 months (29). In another retrospective study, 53 patients with locally advanced or recurrent GBC received immunotherapy as first-line treatment, among whom 10 participants received camrelizumab alone, 22 received camrelizumab plus apatinib therapy, and 21 received camrelizumab plus chemotherapy. The mPFS and mOS of the combination therapy groups, especially the camrelizumab plus chemotherapy group, were greater than those of the single-agent group (camrelizumab plus apatinib group: 6 *vs*. 3 months, P<0.001, 12 *vs*. 8 months, P=0.019; camrelizumab plus chemotherapy group:9 *vs*. 3 months, P<0.001, 13 *vs*. 8 months, P<0.001, respectively) (33). There are very few data on chemotherapy combined with immunotherapy in the second-line treatment of GBC. In a phase II study of pembrolizumab plus capecitabine and oxaliplatin (CapOx) for 11 patients with BTC, 8 of patients had received one or more systemic platinum-based chemotherapies. Three patients with GBC were enrolled, 2 of whom were evaluated for SD and 1 for PR (34).

In our case series, 11 patients received PD-1 inhibitors in combination with nab-paclitaxel-based chemotherapy as second-line therapy. The PFS was 7.5 months, and the median OS was 12.7 months, providing better results in comparison to previous studies. Although such a combination is not currently the standard second-line treatment for GBC, it has been validated to show good antitumor activity and acceptable safety in second-line or postline treatment of breast cancer, urothelial cancer, and gastric cancer (18–20). In our case series, clinicians treated patients based on experience and the patient’s individual situation at the time, with 8 patients treated with nab-paclitaxel monotherapy and 3 patients treated with nab-paclitaxel combined with other cytotoxic agents. Limited by our small number of cases, we did not perform a statistical analysis to explore whether the nab-paclitaxel combination was superior to nab-paclitaxel monotherapy. However, in other tumors, nab-paclitaxel monotherapy in combination with immunotherapy has been studied more frequently as the backline treatment and has shown good efficacy (18–20). Studies have found that nab-paclitaxel may have a synergistic effect in combination with ICIs due to its special nanoparticle carrier (35). The potential mechanisms are associated with promoting the release of tumor-associated antigens, affecting the tumor immune microenvironment, and promoting the accumulation of effector T lymphocytes around the tumor (35).

Accurate screening of patients with high response rates to immunotherapy is an intense area of research. Previous literature reported that the rate of PD-L1 positivity in GBC tumor cells (cutoff 1%) fluctuated from 14.7-23% and was associated with a poorer prognosis (36–39). However, the correlation between PD-L1 and the efficacy of immunotherapy in GBC is less studied. In a few retrospective trials, high PD−L1 expression was associated with a better response to ICIs in advanced BTC; these studies included only a small number of patients with GBC (22, 40). In our cohort, in one patient with positive PD-L1, CR was observed after second-line PD-1 inhibitor plus nab-paclitaxel therapy, which has not been reported in GBC. TMB can predict the efficacy of immunotherapy in many tumor types (41). Most patients with GBC have a low TMB (42). Abdel et al. examined the TMB of 760 patients with gallbladder cancer and showed that the median TMB was 2.6 Muts/Mb, and our cohort’s mTMB was similar to that reported in a previous study (42). In our cohort, 8 patients had available genetic testing, all had MSS, and none of the patients had therapeutically relevant genetic alterations. Biomarkers to predict the efficacy of immunotherapy in patients with advanced GBC need to be further explored.

Several limitations of our exploratory analysis must be acknowledged. First, this is a single-arm exploratory retrospective analysis with a small size and heterogenous patient population. Second, the chemotherapy regimens in this cohort were not completely consistent, which reflected a natural flaw of retrospective studies. In addition, different PD-1 inhibitors were used in this study, which was determined by the economic status of the patients. Although the mechanisms associated with these inhibitors were previously reported to be similar, bias could still occur in a strict sense, during clinical use. A well-designed prospective randomized clinical trial with a controlled arm is needed to address the above issues.

In summary, our results reveal that PD-1 inhibitors combined with nab-paclitaxel-based chemotherapy as second-line therapy might be valuable in treating advanced GBC patients. An exploratory prospective clinical trial is underway at our center (ChiCTR2100052118). We looked forward to seeing more large randomized controlled prospective cohorts to establish the benefits of this combination in GBC patients.

## Data availability statement

The original contributions presented in the study are included in the article/Supplementary Material. Further inquiries can be directed to the corresponding author.

## Ethics statement

The studies involving human participants were reviewed and approved by the ethics committee on biomedical research, West China Hospital of Sichuan University. The patients/participants provided their written informed consent to participate in this study.

## Author contributions

ST collected the data and wrote the manuscript. JY, QH, and NZ helped collect literature and participated in discussions. HG designed the study and examined the language. All authors contributed to the article and approved the submitted version.

## Funding

This work was supported by the Health Commission of Sichuan Province Program (21PJ007).

## Acknowledgments

We would like to thank the patients in this study and all staff at the hospital for their contributions to this study.

## Conflict of interest

The authors declare that the research was conducted in the absence of any commercial or financial relationships that could be construed as a potential conflict of interest.

## Publisher’s note

All claims expressed in this article are solely those of the authors and do not necessarily represent those of their affiliated organizations, or those of the publisher, the editors and the reviewers. Any product that may be evaluated in this article, or claim that may be made by its manufacturer, is not guaranteed or endorsed by the publisher.
